# A progressive declining in the burden of malaria in north-eastern Tanzania

**DOI:** 10.1186/1475-2875-9-216

**Published:** 2010-07-23

**Authors:** Bruno P Mmbando, Lasse S Vestergaard, Andrew Y Kitua, Martha M Lemnge, Thor G Theander, John PA Lusingu

**Affiliations:** 1 National Institute for Medical Research, Tanga Centre, Tanga, Tanzania; 2 Department of Biostatistics, University of Copenhagen, Copenhagen, Denmark; 3 Centre for Medical Parasitology at Department of International Health, Immunology and Microbiology, University of Copenhagen, and Department of Infectious Diseases, Copenhagen University Hospital (Rigshospitalet), Copenhagen, Denmark; 4 Special Programme for Research & Training in Tropical Diseases (TDR), World Health Organization, Geneva, Switzerland

## Abstract

**Background:**

The planning and assessment of malaria interventions is complicated due to fluctuations in the burden of malaria over time. Recently, it has been reported that the burden of malaria in some parts of Africa has declined. However, community-based longitudinal data are sparse and the reasons for the apparent decline are not well understood.

**Methods:**

Malaria prevalence and morbidity have been monitored in two villages in north-eastern Tanzania; a lowland village and a highland village from 2003 to 2008. Trained village health workers treated presumptive malaria with the Tanzanian first-line anti-malarial drug and collected blood smears that were examined later. The prevalence of malaria parasitaemia across years was monitored through cross-sectional surveys.

**Results:**

The prevalence of malaria parasitaemia in the lowland village decreased from 78.4% in 2003 to 13.0% in 2008, whereas in the highland village, the prevalence of parasitaemia dropped from 24.7% to 3.1% in the same period. Similarly, the incidence of febrile malaria episodes in the two villages dropped by almost 85% during the same period and there was a marked reduction in the number of young children who suffered from anaemia in the lowland village.

**Conclusion:**

There has been a marked decline in malaria in the study villages during the past few years. This decline is likely to be due to a combination of factors that include improved access to malaria treatment provided by the trained village helpers, protection from mosquitoes by increased availability of insecticide-impregnated bed nets and a reduced vector density. If this decline in malaria morbidity is sustained, it will have a marked effect on the disease burden in this part of Tanzania.

## Background

There have been previous efforts to eradicate or, if this was not possible, to eliminate malaria, but these ambitious goals have not been achieved in large parts of Africa [1-5] despite the development of effective tools, such as insecticide-impregnated bed nets (ITNs) and case management on demand; and malaria vaccines are being developed to complement the existing tools [6-9]. The performance of these tools can be monitored by careful surveillance of the malaria burden in a defined community before and after their deployment [10-12]. However, malaria transmission and the associated disease burden can fluctuate in the absence of any intervention due to climatic variations and societal behavioural changes. These changes can be dramatic in areas of unstable and seasonal malaria transmission, but are much less pronounced in areas of high and stable transmission [13,14]. In the past few years, reports have documented that the malaria burden has diminished in several areas of Africa [15-18], but the reasons for these changes are still uncertain. This fortunate development has been ascribed to a combination of interventions, such as use of ITNs, artemisinin combination therapy (ACT) and indoor residual spraying (IRS) [16]. This paper documents a marked decline in the malaria burden in two Tanzanian villages in an area with stable malaria transmission during the period 2003 to 2008. The decrease was most marked in a village where transmission was high at the beginning of the study and took place before the introduction of ACT.

## Methods

### Study area

The study was conducted in Korogwe District, north-eastern Tanzania. Korogwe District is about 100 kilometers inland from the coastal town, Tanga. It is a tropical area with two rainy seasons: April to June and October to December. January and February are normally dry, but recently there have been climatic changes with merging of these two rainy seasons. Entomological surveys in the study area have shown that *Anopheles gambiae *is the most prevalent vector in the lowlands, while *Anopheles funestus *predominates in the highlands. In previous studies, entomological inoculation rates (EIR) have been reported to be more than 100 in the lowlands, but less than 30 infective bites per person per year at intermediate altitudes. In the highlands, mosquito densities are too low to allow reliable EIR measurements, but an EIR of 0.03 infective bites per person per year has been extrapolated [19]. There is no recent entomology data for the area, the most recent were collected in the late 1990 s. *Plasmodium falciparum *is the dominant malaria parasite with *Plasmodium malariae *being encountered occasionally. Villagers perceive malaria as a major health problem among both children and adults leading to disease and deaths.

The study was conducted in two villages, Mkokola and Kwamasimba, which had 2,000 and 1,800 inhabitants, respectively in 2003. Mkokola village is situated at an altitude of about 300 meters above sea level (lowland village) and Kwamasimba at an altitude of approximately 700 meters (highland village). The two villages are approximately 15 kilometres apart [20-22].

### Longitudinal detection of febrile episodes

In January 2003, management of uncomplicated malaria cases by trained village health workers was introduced into the study villages. The village health workers, locally called Community Owned Resource Persons (CORPS) carried out passive case detection of subjects with fever. There were two CORPS in each village, one of whom was always present in the village, so that any village resident who had a febrile illness, had an opportunity to obtain treatment at any time. The CORPS were provided with thermometer, first-line anti-malarial drugs (sulphadoxine-pyrimethamine [SP] up to 2006 and artemether-lumefantrine [ALu] from 2007 to date), paracetamol, slides, blood lancets, gloves, treatment charts, febrile case detection forms and storage boxes. CORPS were instructed how to collect blood smears under aseptic technique and to preserve these until collected by a team of supervisors composed of a clinical officer and a laboratory technician. CORPS were also instructed on how to treat patients with the first-line anti-malarial drug and an antipyretic, and how to refer patients who had severe symptoms or who did not respond adequately to the first-line anti-malarial treatment to Korogwe District Hospital. Villagers were informed that they could call on the CORPS at any time if they had symptoms of malaria. From suspected malaria cases, the CORPS would then collect basic clinical data, obtain a blood smear for malaria microscopy and institute presumptive malaria treatment. Patients with severe symptoms or symptoms suggestive of other diseases than malaria were referred to the local dispensary or the District Hospital.

### Cross-sectional surveys

Malaria cross-sectional surveys were conducted in the two study villages from 2003 to 2008 (Table 1). The surveys were conducted in May during the main rainy season in the years 2003-2008, and just before the short rains in November in the years 2003-2007. During each cross-sectional survey, demographic data were collected together with a history of migration and recent travel. The reported use of a bed net was also recorded. A history of recent illness was obtained, emphasizing symptoms suggestive of malaria. Physical examination for signs related to malaria, such as temperature, pulse, spleen size, pallor or a raised respiratory rate was conducted. Axillary temperature was measured using digital thermometers. For any individual diagnosed with a mild disease, appropriate drugs were administered.

**Table 1:** Number of participants surveyed per village per year

Village	Age group (years)	Number of participants surveyed per year					
		2003	2004	2005	2006	2007	2008
**Lowland village**	**<1**	30	15	20	30	23	5
	**1**	35	48	55	34	35	21
	**2**	31	63	61	52	14	20
	**3**	29	45	79	52	20	7
	**4**	26	47	63	70	23	20
	**5-9**	76	157	191	212	130	59
	**10-14**	47	65	153	56	165	49
	**15-19**	36	52	55	18	58	22
	
	**Total**	**310**	**492**	**677**	**524**	**468**	**203**

**Highland village**	**<1**	25	23	42	33	34	5
	**1**	25	51	80	50	44	14
	**2**	20	53	73	72	40	17
	**3**	29	32	77	47	60	15
	**4**	28	56	58	67	48	20
	**5-9**	81	160	123	220	207	55
	**10-14**	44	63	75	90	141	52
	**15-19**	31	54	46	28	40	34
	
	**Total**	**283**	**492**	**574**	**607**	**614**	**212**

Five millilitres of venous blood were collected from individuals aged three years or more. For children below three years, 300-400 μl of capillary blood was obtained by finger prick into an Eppendorf tube containing EDTA. The haemoglobin (Hb) concentration of each participant was measured using a HemoCue^® ^photometer (Ångelholm, Sweden). Whole blood was used to prepare thick and thin blood smears for malaria microscopy. These were stained with 10% Giemsa stain for 15-20 minutes after fixing thin smears with methanol. Asexual and sexual parasites were counted against 200 and 500 white blood cells, respectively. The differentiation of malaria parasite species was confirmed by microscopy of thin smears. A blood smear was declared negative only after examination of 200 high power fields. The density of asexual parasites was calculated assuming 8,000 leucocytes per μl of blood and expressed as parasites per μl. Slides were red retrospectively and during surveys malaria treatment was only given to patients complaining of malaria symptoms.

### Data management and analysis

All data were double-entered into a Microsoft Access database, cleaned, validated and transferred into Stata version 9 (Stata Corporation, Texas, USA) and R 2.7.0 for statistical analysis. Fever was defined as an observed axillary temperature of ≥37.5°C and/or a history of fever within the previous 24 hours. A febrile malaria episode was defined as an episode of fever with a positive blood smear for asexual *Plasmodium falciparum *parasitaemia of any density. The summary measures consisted of percentage for categorical variables and means for continuous variables. Incidence was calculated as documented number of febrile malaria episodes to the population size in each age group and/or village per year.

In modelling the risk of *Plasmodium falciparum*, parasiteamia across the years, number of individuals with parasitaemia during cross-sectional surveys were modelled as a binomial random variable using logistic regression. The model was fitted for each village separately and was adjusted for the effect of age and use of bednets. To compare the risk of *Plasmodium falciparum *infection between the two villages, a similar model was fitted for the two villages. In addition, linear regression models were used to determine the changes in mean haemoglobin (Hb) concentration across years and effect of *Plasmodium falciparum *parasiteamia and season on Hb levels while controlling for age and reported use of insecticide-treated or non-treated bed nets. In this model the effect of year was fitted as a categorical variable. A statistical significance level was considered at P-value < 0.05.

### Ethical considerations

Ethical clearance was granted by the Medical Research Co-ordinating Committee of the National Institute for Medical Research, Tanzania. Prior to the study, meetings were held with local authorities and with the villagers in each study village, during which the aims of the study were explained. Informed consent documents for the study were prepared in English and translated into Kiswahili before administration to both village leaders and participants. Written informed consent to participate was obtained from each study individual or from his or her parent or guardian. Villagers were free not to participate in the study without giving any reasons, or being disqualified from any medical services that were provided to all villagers throughout the study period. Feedback to the village has been performed continuously during the study.

## Results

### Malaria parasitaemia by year

**Figure 1:**
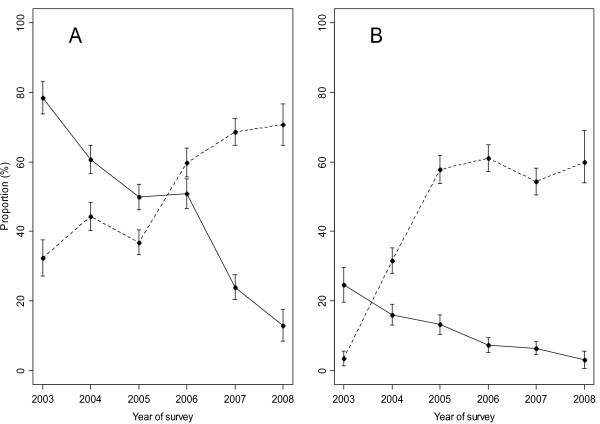
**Proportion of *Plasmodium falciparum *parasitaemia and reported use of insecticide treated or non-treated nets obtained during cross-sectional surveys**. Filled circles connected with solid lines for the proportion of *Plasmodium falciparum *and filled circles with dotted lines for proportion of reported use of insecticide treated or non-treated nets. Panel A for lowland village and Panel B for highland village.

**Figure 2:**
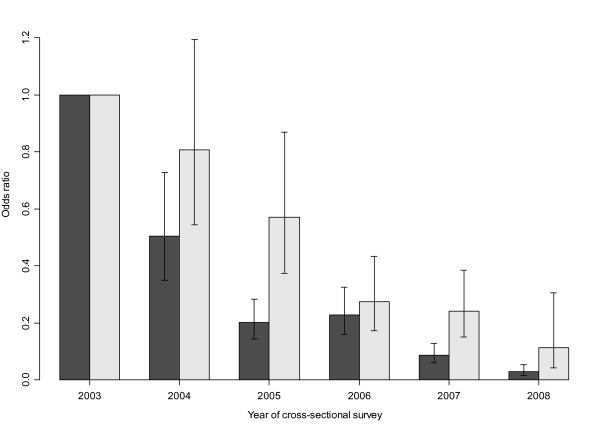
**Odds ratio of *Plasmodium falciparum *parasite adjusted for age, bed nets use and season of survey from 2003-2008, with 2003 as a baseline**. Filled and open bars are for lowland and highland villages, respectively.

**Figure 3:**
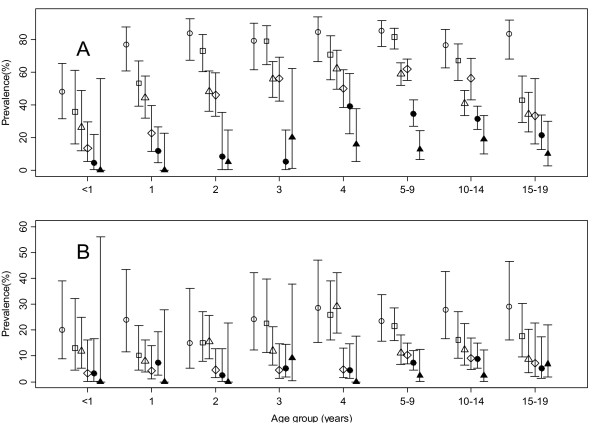
**Age-specific point prevalence of *Plasmodium falciparum *and 95% CI (line segments) obtained during cross-sectional surveys between 2003 and 2008 in lowland village (panel A) and highland village (panel B)**. Symbol representing the year of the survey, open circles (2003), open squares (2004), open triangles (2005), open diamond (2006), filled circles (2007) and filled triangles (2008).

At the first cross-sectional survey, *P. falciparum *was detected in 78% and 25% of the residents of lowland and highland villages, respectively. In the subsequent cross-sectional surveys conducted each year, the prevalence of *P. falciparum *decreased steadily in both villages as shown Figures 1 and 2. The risk of *P. falciparum *infection was higher in residents of lowland village, OR = 12.28 (95%CI: 9.44 - 15.10, p < 0.001) compared to highland village. The risk of infection decreased by year, with an OR of 0.564 (95% CI: 0.526-0.605, p < 0.001) and 0.660 (95% CI: 0.602-0.730, p < 0.001), per year compared with the baseline, for lowland and highland villages, respectively. The decline in the point prevalence of parasiteamia was seen in all age groups in both villages (Figure 3), but was most pronounced among one to three year old children in the high transmission village (lowland village). During the study, reported use of both impregnated nets and/or non-impregnated bed net increased from 32% to about 80% in the high transmission village (lowland village) and from 4% to about 60% in the low transmission village (highland village).

Use of a bed nets was associated with a reduction in the risk of parasitaemia OR = 0.666 (95%CI: 0.575 - 0.770, p < 0.001). When the effect of the use of bed nets was assessed in each village separately, it was found that the use of bed nets was not associated with a reduction in the risk of malaria parasitaemia in highland village (OR = 0.921 (95%CI: 0.708 - 1.198, p = 0.540), but was significant in lowland village (OR = 0.572 (95%CI: 0.479 - 0.681, p < 0.001).

### Incidence of febrile malaria episodes by age

**Figure 4:**
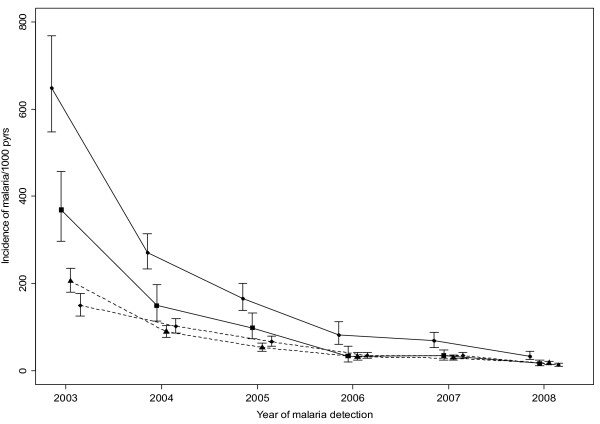
**The incidence of febrile malaria episodes among residents of lowland village (filled circles and triangles) and highland village (filled squares and diamonds) between 2003 and 2008**. Full lines represent children < 5 years, while dashed lines are individuals aged ≥ 5 years.

The age specific incidence of malaria episodes by village is shown in Figure 4. Children below five years in lowland village had a higher incidence than children in highland village throughout. The incidence of episodes of malaria (per 1,000 person years) among children under five years of age declined dramatically in both villages during the 2003-2008 study period from 649.0 (95%CI:548.3 - 768.3) to 33.6 (95%CI:25.5 - 44.4), and 368.9 (95%CI:297.5 - 457.4) to 16.8 (95%CI:11.7 - 24.0) in lowland and highland villages, respectively. The decrease in incidence was most marked during the first three years of the study and there was only a minor difference in incidence between 2006 and 2008. This was the case in individuals of all ages and in both villages.

### Haemoglobin concentration by year

The mean haemoglobin concentration of individuals living in highland village was higher than that of those living in lowland village. In highland village, mean haemoglobin concentration was highest in 2003, whereas in lowland village it was the lowest in this year. Similarly, the mean haemoglobin concentration of children aged 0-3 from lowland village was markedly lower in 2003 than in the following years (Figure 5). Adjusting for effect of age and sex, there was a trend to increasing mean haemoglobin concentration in lowland village by year (Table 2). Contrarily, the mean haemoglobin was significantly lower in highland village in all surveys conducted in 2004-2008 compared to one in 2003, (Table 2 and Figure 5). In lowland village, use of bed nets had no apparent effect on mean haemoglobin concentration while in highland village it was associated with a significantly increase in mean haemoglobin by 0.149 g/dl. In both villages, the presence of *Plasmodium falciparum *parasitaemia was associated with a lower mean haemoglobin concentration (Table 2).

**Table 2:** Linear regression results showing the change in mean haemoglobin concentration (g/dL) in highland and lowland villages by year and other variables when adjusted for age and gender

	Lowland village			Highland village		
		
Variable	Coefficient	(95%CI)	P-value	Coefficient	(95%CI)	P-value
Intercept	9.253	(8.822 - 9.684)	<0.001	11.073	(10.652 - 11.494)	<0.001
Use of net	0.039	(-0.075 - 0.153)	0.495	0.149	(0.031 - 0.267)	0.013
*P. falciparum^§^*	-0.388	(-0.506 - -0.27)	<0.001	-0.471	(-0.642 - -0.300)	<0.001
Survey period^#^	-0.28	(-0.400 - -0.160)	<0.001	-0.254	(-0.374 - -0.134)	<0.001
Year 2003	Reference					
Year 2004	0.145	(-0.065 - 0.355)	0.175	-0.933	(-1.154 - -0.712)	<0.001
Year 2005	0.327	(0.129 - 0.525)	0.001	-1.199	(-1.42 - -0.978)	<0.001
Year 2006	0.286	(0.076 - 0.496)	0.008	-1.248	(-1.469 - -1.027)	<0.001
Year 2007	0.134	(-0.087 - 0.355)	0.235	-1.048	(-1.269 - -0.827)	<0.001
Year 2008	0.424	(0.130 - 0.718)	0.005	-0.515	(-0.821 - -0.209)	<0.001

§ Blood slide positivity for *Plasmodium falciparum *at sampling

# Effect of blood sample collected in November versus May

**Figure 5:**
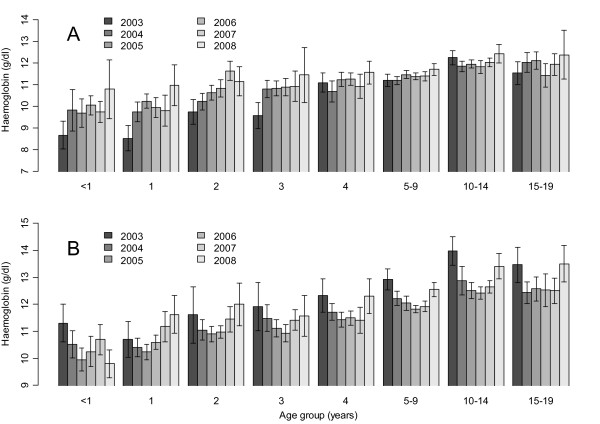
**Age-specific mean haemoglobin concentration (g/dl) measured during cross-sectional surveys between 2003 and 2008 in lowland (A) and highland (B) villages, respectively**. Graduated grey colour shows year of survey and line segments represent 95% CI.

## Discussion

This community based study assessed the malaria burden in two villages in Korogwe district, north-eastern Tanzania by collecting data in repeated cross-sectional surveys and by employing CORPS to monitor malaria morbidity over six consecutive years. In this study, lowland residents had almost a twelve-fold increase in the risk of malaria parasitaemia compared to their counterparts in the highland village. At the start of the study, the lowland village could be categorized as high transmission whereas transmission in the highland village could be categorized as moderate. During the six years of the study, there was a marked and progressive decline in the malaria burden and this was reflected in the point prevalence of parasitaemia, the incidence of clinical episodes of malaria and in the mean haemoglobin concentration.

It is well established that immunity to malaria is a function of age, and that in areas of high transmission the malaria burden mainly is carried mainly by children and infants, while in areas of low transmission, all age groups are affected [23]. As malaria transmission declined in the study area, a change in the age distribution of the malaria burden was detected. In 2003, young children resident in the high transmission village had a markedly higher incidence of malaria fevers than older children and young adults, and many of these children were anaemic. Indeed, the most striking finding of this study was the pronounced increase in the haemoglobin concentrations among 1-3 year old children in the lowland village. This represents a remarkable health gain and underscores findings of previous studies showing the high impact malaria has on haemoglobin levels in young children in areas of high transmission [15,24,25]

During the study, the malaria burden also decreased among inhabitants of highland village and among the older age groups in lowland village, but because the decline in burden was more pronounced among the young children in lowland village, the net effect was that, at the end of the study the malaria burden appeared to be more equally distributed among the different age groups. During the study, many cases of severe malaria were not documented. This is probably due to the fact that a relatively low number of individuals were surveyed, and that these individuals had access to prompt and effective treatment. Thus, how the decline in transmission has affected the incidence of severe malaria in its different manifestations could not be estimated. This, is an important question as cerebral malaria has been reported to affect slightly older children than those affected by severe anaemia, and a declining incidence might lead to an increase in this severe form of malaria [14,26].

Other studies mainly based on data collected at health facilities have documented that malaria transmission seems to be falling in other parts of East Africa and in some of these studies the decrease has been attributed to specific interventions [14-18]. In this community based study, a dramatic decrease in malaria indices in the absence of specific interventions other than giving villagers access to treatment through trained village helpers is documented. During the first four years of the study period, the first-line anti-malarial was SP, but from January 2007 an ACT was used. Thus, the most dramatic decrease in malaria burden took place when SP, a drug to which there is widespread resistance in the area, was in use [27]. Although the presumptive treatment of febrile cases could have played a role in reducing transmission[28], this could not explain the findings. In 2005, World Vision deployed many nets in Korogwe, including the study villages. Although the coverage was not complete, there has been a substantial increase in use of nets in the study villages, approaching 60%. It has been argued that communal effect of protection is achieved when coverage reaches 64%, which is almost the case in these villages [26], but again whether nets at the coverage reached could have had such pronounced effects on transmission as indicated by the decrease in point prevalence from 2003 to 2008 is still debatable. As in Tanzania in general, there has been a steady socio-economic development in the area, but there have not been major changes or investments in infrastructure in the two study villages that can explain the decrease in malaria morbidity.

There is no simple explanation for the decline in malaria in the study villages, and it is rather an unsatisfactory situation of having to assign this to a combination of different factors such as climatic changes, better health provision, increased coverage of bed nets and socio economical development. It is very important to establish the relative contribution of these and other factors to the decline in malaria, as this will shed light on the risk that malaria might strike back. Entomological studies in the study villages were not done during the study period and whether there have been changes in mosquitoes distribution across years is not established.

In conclusion, there has been a dramatic progressive decline in point prevalence of malaria in the study villages during the study period, and in parallel the malaria disease burden has fallen, especially among those most affected previously, the infants and young children living in the village where malaria transmission was high previously.

## Competing interests

The authors declare that they have no competing interests.

## Authors' contributions

All authors were involved in the design of the study. LV, BM, AK, ML, TT and JL conceived the study. BM, LV and JL and were responsible for the implementation of the study in the field. BM coded the data and supervised data entry. BM and JL analysed data. JL wrote the first draft of the manuscript. All authors were involved in revising the manuscript, and read and approved the final version.
